# The Case of Emperor Frederick III

**Published:** 1889-04

**Authors:** 


### Book Notices.

THE CASE OF EMPEROR FREDERICK III. Full official reports by the German physicians and by Sir Morell MacKenzie. Translated and both sides reviewed by Henry Schweig, M. D., Laryngologist, New York. Cloth $1 25, paper 75 cents. Edgar S. Werner Publisher, New York.

The book contaius 276 pages, and is said to be the only edition giving the uuabridged reports, with all of the illustrations of Sir Morell MacKenzie and the German physicians.

While it is rather interesting reading, the book contains a mass of irrelevant matter that will tire one before he has finished reading it.

Both reports exhibit a great deal of personal spite and jealousy. The result was great notoriety to all concerned but no credit to the profession. B.

